# Impact of Glutathione S-Transferase M1 and T1 on Anti-Tuberculosis Drug–Induced Hepatotoxicity in Chinese Pediatric Patients

**DOI:** 10.1371/journal.pone.0115410

**Published:** 2014-12-19

**Authors:** Fang Liu, An-xia Jiao, Xi-rong Wu, Wei Zhao, Qing-qin Yin, Hui Qi, Wei-wei Jiao, Jing Xiao, Lin Sun, Chen Shen, Jian-ling Tian, Dan Shen, Evelyne Jacqz-Aigrain, A-dong Shen

**Affiliations:** 1 Key Laboratory of Major Diseases in Children and National Key Discipline of Pediatrics (Capital Medical University), Ministry of Education, Beijing Pediatric Research Institute, Beijing Children's Hospital, Capital Medical University, Beijing, China; 2 Department of Clinical Pharmacy, School of Pharmaceutical Sciences, Shandong University, Jinan, Shandong, China; 3 Department of Pediatric Pharmacology and Pharmacogenetics, Hôpital Robert Debré, APHP, Paris, France; 4 Clinical Investigation Center CIC1426, INSERM, Paris, France; 5 EA7323, Université Paris Diderot-Université Paris Descartes, Paris, France; Nottingham University, United Kingdom

## Abstract

**Background:**

Anti-tuberculosis drug induced hepatotoxicity (ATDH) is a major adverse drug reaction associated for anti-tuberculosis therapy. The glutathione S-transferases (GST) plays a crucial role in the detoxification of hepatotoxic metabolites of anti-tuberculosis drugs.An association between GSTM1/GSTT1 null mutations and increased risk of ATDH has been demonstrated in adults. Given the ethnic differences and developmental changes, our study aims to investigate the potential impacts of GSTM1/GSTT1genotypes on the development of ATDH in Han Chinese children treated with anti-tuberculosis therapy.

**Methods:**

Children receiving anti-tuberculosis therapy with or without evidence of ATDH were considered as the cases or controls, respectively. The GSTM1 and GSTT1 genotyping were performed using the polymerase chain reaction.

**Results:**

One hundred sixty-three children (20 cases and 143 controls) with a mean age of 4.7 years (range: 2 months-14.1 years) were included. For the GSTM1, 14 (70.0%) cases and 96 (67.1%) controls had homozygous null mutations. For the GSTT1, 13 (65.0%) cases and 97 (67.8%) controls had homozygous null mutations. Neither the GSTM1, nor the GSTT1 polymorphism was significantly correlated with the occurrence of ATHD.

**Conclusion:**

Ourresults did not support the GSTM1 and GSTT1 polymorphisms as the predictors of ADTH in Chinese Han children treated with anti-tuberculosis drugs. An age-related association between pharmacogenetics and ATHD need to be confirmed in the further study.

## Introduction

Tuberculosis is a threat to worldwide public health. Globally, about a third of the world's population is infected by the tuberculosis bacteria. In 2009, there were an estimated 9.4 million incident cases (equivalent to 137 cases per 100 000 population) of tuberculosis [1]. Among 22 countries with the highest burden of tuberculosis, China is the second highest after India. According to the Chinese national epidemiological survey, the infection rate of tuberculosis bacteria was around 44.5% in the whole population and 9.0% in children below 14 years. The active tuberculosis rate was about 91.8 cases per 100 000 population [2].

Anti-tuberculosis drug therapy plays an important role to control tuberculosis epidemic in children [3]. The standard pediatric anti-tuberculosis therapy includes a combination of isoniazid, rifampicin, pyrazinamide and ethambutol. Anti-tuberculosis drug induced hepatotoxicity (ATDH) is a major adverse drug reaction associated for anti-tuberculosis therapy [4], [5]. The reported incidence of ATDH ranged from 2.46% to 32.1% [6], [7]. Asymptomatic increase of transaminases is the most common clinical manifestation of ATDH, but lethal hepatic failure may also occur when treatment is not interrupted in time [8]. ATHD is a multifactorial disorder and different risk factors have been identified, e.g. ethnic, age, sex, pre-existing liver disease etc. [9]–[13].

In recent years, the pharmacogenetic research has received much attention to identify the genetic predictors of ATDH in order to individualize anti-tuberculosis therapy. Researches on the glutathione S-transferases (GST) genes have obtained promoting results to identify/predict special patient at risk of ATDH. The GST genes code for a superfamily of enzymes that are involved in the phase-II drug metabolism andplay a crucial role in the biological detoxification processes of many drugs including anti-tuberculosis drugs. They catalyze the conjugation reactions of glutathione and toxic intermediary metabolites and facilitate toxicant elimination, thereby, decreasing the risk of the drug-induced hepatotoxicity. The recent meta-analysis, which involved 13 case-control studies and more than 900 ATHD cases, has demonstrated that the GSTM1 homozygous null genotype was associated with an increased risk of ATDH [14]. Moreover, this association seems to be ethnic-dependent. East Asian patients with GSTM1 null genotype had a higher risk than Caucasians [15], Whereas, GSTT1 homozygous null genotype was a risk factor of ATHD in Caucasians [16], but not in Chinese [17].All of the published studies were conducted in adults. There was no study conducted in children. Given the ethnic difference in the correlation between the GST genetic polymorphism and ATDH, and apotential impact of age, our study aims to investigate the impacts of the GSTM1 and GSTT1 genotypes on the development of ATDH in Han Chinese children receiving anti-tuberculosis therapy.

## Materials and Methods

### Study design and patients

A total of 223 unrelated active tuberculosis patients, who were treated with standard anti-tuberculosis protocol between 2005 and 2010, were enrolled in this study at the Tuberculosis Ward, Beijing Children's Hospital, Capital Medical University (Beijing, China). The standard anti-tuberculosis therapy protocol consists of isoniazid(INH,H) 10–20 mg/kg/day (up to a maximum of 300 mg/day), rifampicin(RFP,R) 10–20 mg/kg/day (up to a maximum of 450 mg/day), pyrazinamide(PZA,Z)20–30 mg/kg/day(up to a maximum of 1500 mg/day), ethambutol (EMB, E) 15–25 mg/kg/day, and streptomycin(SM,S) 20–30 mg/kg/day (up to a maximum of 750 mg/day). For primary complex and infiltrative pulmonary tuberculosis, INH and RFP is used for 6–9 months; for tuberculosis of bronchial lymph nodes merge of bronchial tuberculosis, infiltrative pulmonary tuberculosis with cavity and spread of bronchogenic, the treatment is started with INH/RFP/PZA for 3 months and followed by INH/RFP for 3–6 months; For miliary tuberculosis and caseous pulmonary tuberculosis, the treatment is started with INH/RFP/PZA/SM for 2 months, followed by INH/RFP/PZA for 1 month and INH/RFP for 6 months, and then, according to patient's clinical condition to decide if continues with INH for 3 months. For disseminated tuberculosis, the treatment is started with INH/RFP/PZA for 3 months, (SM can be added in serious case), followed by INH/RFP for 6–9 months. For tuberculous meningitis, the treatment is started with INH/RFP/PZA for3 months(SM/EMB can be added), and followed by INH/RFP for 9 months (or 6 months after cerebrospinal fluid cell count and biochemical became normal).

This study was approved by the Ethical Committee of the Beijing Children's Hospital and written informed consents were obtained from all of the enrolled children or their parents/guardians. Patients who meet the following inclusion criteria were eligible for the study: (i) Chinese Han children aged between 0 and 16 years; (ii) diagnosis of active tuberculosis by clinical examination, radiological and microbiological investigations; (iii) standard anti-tuberculosis treatment has been started for at least two weeks; (iv) serum transaminases were normal before treatment (alanine aminotransferase ALT<40 IU/L, aspartate aminotransferase AST<40 IU/L). Patients with pre-existing liver disease, viral hepatitis, chronic alcoholism, or history of intake of other hepatotoxic drugs were excluded from the study.

In this case-control study, the case was selected from children with ATDH and fulfilled the inclusion criteria mentioned above. The diagnostic criteria of ATDH was based on the international consensus [18]–[20]: (i) serum ALT>2×ULN (upper limit of normal, 40 IU/L); or (ii) serum direct bilirubin (DBil)>2×ULN (6.8 µmol/L); or (iii) increases of serum AST (40 IU/L), alkaline phosphatase (ALP,220 IU/L) and total bilirubin (TBil, 19.0 µmol/L), moreover, one of them>2×ULN; or (iv) any index mentioned above>1×ULN and associated with liver damage symptoms, such as skin or sclera yellow dye, severe anorexia, nausea, vomiting, fever, rash, itching. Conform to anyone of above four is defined as liver damage, and liver damage occurred after receiving anti-tuberculosis therapy and was relieved when the dose of tuberculosis drugs was reduced or treatment was stopped.

Controls were defined as patients who fulfilled the inclusion criteria but did not have any symptoms and/or laboratorial evidence of liver dysfunction during the anti-tuberculosis therapy. Controls were matched to cases on the basis of age, sex and anti-tuberculosis therapy.

**Figure 1:**
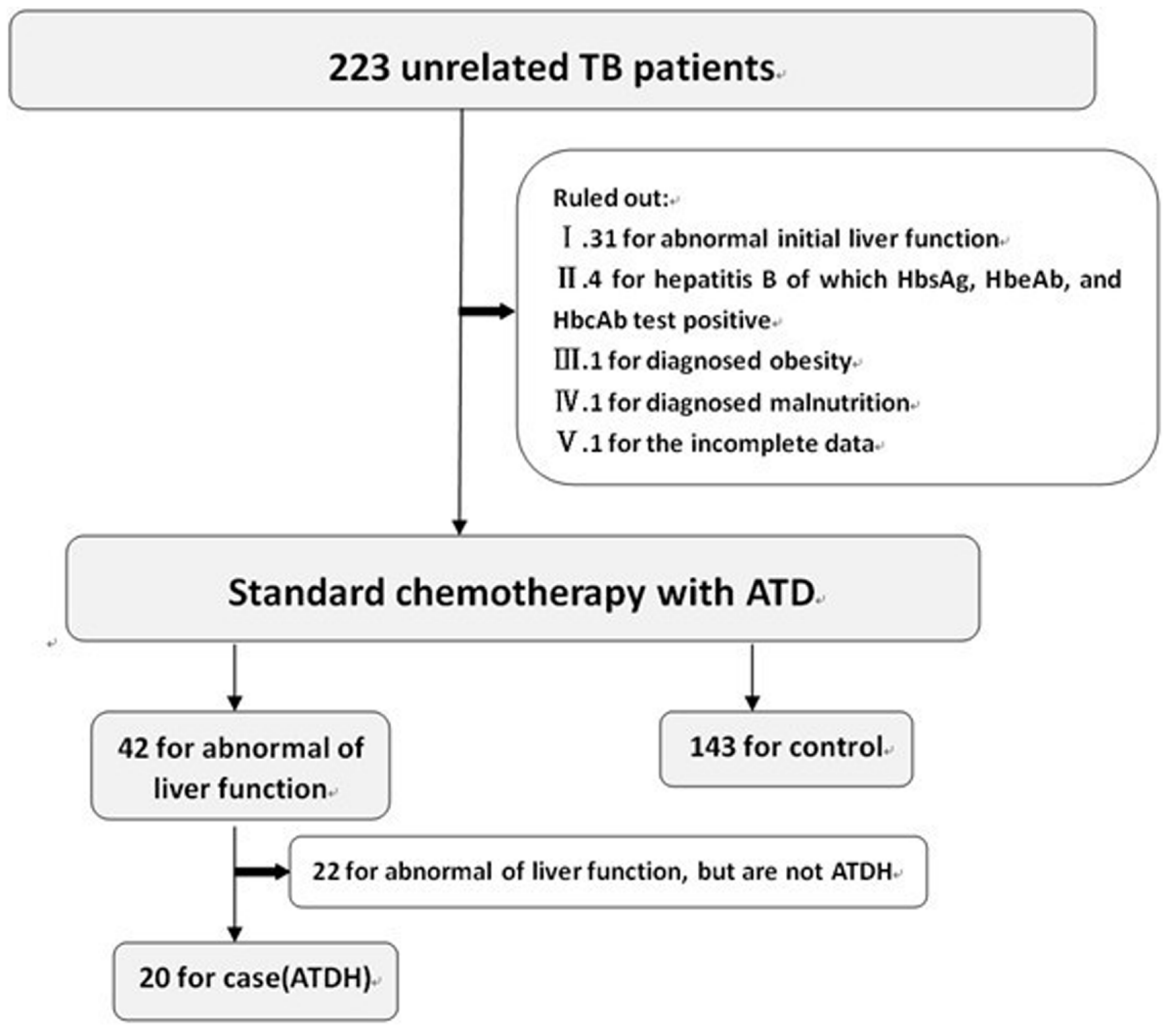
The flow chart of case-control study. A total of 223 unrelated active tuberculosis patients treated with standard anti-tuberculosis protocol between 2005 and 2010. According to inclusion criteria, we got 20for case and 143 for control.

The flow chart of our study is presented in fig. 1. Overall, 223 tuberculosis children were screened, 60 were excluded and 163 were included in the final analysis (20 for case and 143 for control). For 20 cases, 1 patient was treated with INH, 1 patient with INH/RFP, 15 patients with INH/RFP/PZA, and 3 patients with INH/RFP/PZA/EMB.

According to the most recently published study in adults, the significant impacts of GSTM1 and GSTT1 null mutations on the risk of ATHD have been demonstrated in 17 ATHD patients with an odds ratio (OR) of 3.59 [21]. Using this OR, 20 ATHD children, included in the present study, is sufficient to reach a statistical significance (*P*<0.05) for a 1∶7 mating case-control study with a risk alpha of 0.1 and a power of 0.8, based on Chi-square with Yates continuity correction method.

### GSTM1 and GSTT1 genotyping

The isolation of genomic DNA was carried out from peripheral blood mononuclear cells using a standard procedure. The GSTM1and GSTT1 polymorphisms were determined using a multiplex PCR protocol. The primers as previously described [22] with minor modifications are shown in Table 1. PCR was performed in a final volume of 25 µl. The reaction condition of “GSTM1 gene/GSTT1 gene” was: 3 min at 94°C for degeneration, and then 30 cycles (30 s at 94°C, flowed by 30 s at 65°C/57°C and 1 min at 72°C, and finally 5 min at 72°C). The reaction condition of “GSTM1 deletion/GSTT1 deletion” was: 3 min at 94°C for degeneration, and then 40 cycles (10 s at 98°C, flowed by 5 s at 58°C/68°C and 10 min at 72°C, and finally 5 min at 72°C). GSTM1 and GSTT1 genotypes were detected by the absence or presence of a band of PCR product in a 1.5% agarose gel (containing 0.5 µg/ml ethidium bromide), which was visualized by UV light and compared with the molecular weight marker.

**Table 1:** PCR primers for GSTM1 and GSTT1.

primer	Forward primer	Reverse primer	working concentration (µmol·L-1)	annealing temperature (°C)	target fragment (bp)
GSTM1 gene	5′-CAAATTCTGGATTGTAGCAGATCATGC-3′	5′-CACAGCTCCTGATTATGACAGAAGCC-3′	0.2	65	625
GSTM1 deletion	5′-AAGACAGAGGAAGGGTGCATTTGATA-3′	5′-ACAGACATTCATTCCCAAAGCGACCA-3′	0.4	58	4748
GSTT1 gene	5′-TCTTTTGCATAGAGACCATGACCAG-3′	5′-CTCCCTACTCCAGTAACTCCCGACT-3′	0.2	57	969
GSTT1 deletion	5′-GAAGCCCAAGAATGGGTGTGTGTG-3′	5′-TGTCCCCATGGCCTCCAACATT-3′	0.2	68	3106

### Statistical analysis

Data are expressed as the mean ± standard deviation (for normal distribution) or as the median with range (for non-normal distribution). Quantitative variables were analysed using Mann-Whitney U-test. Genotypic frequencies of GSTM1 and GSTT1 were compared between cases and controls using a chi-squared (2×2 table) test with Yates' correction. Analyses were performed using SPSS version 13.0(SPSS Inc., Chicago, IL). A value of *P*<0.05 was considered statistically significant.

## Results

In our study, there were no significant differences in age, sex and liver function at baseline (before treatment) between the cases and controls. As expected, the medians of ALT and AST during treatment were significantly higher in the cases as compared to the controls. In the cases, ATDH occurred in the initial phase of drug therapy (median of 16 days, range 7–26 days), and ALT level returned rapidly to normal value after stopping the treatment (median of 18 days, range 7–39 days) (Table 2).

**Table 2:** Clinical characteristics of study subjects.

	Presence (n = 20)	Absence (n = 143)	*P*
Age (year) (mean ± standard deviation)	3.59±4.24	6.12±4.61	0.138
Sex (M/F)	12/8	85/58	1.000
Baseline [median (range)]			
AST	32.5 (13–84)	26(9–72)	0.140
ALT	22(5–57)	15(5–42)	0.058
TBil	5.8(2.7–16.7)	7.9(1.2–25)	0.067
ALP	154 (70–263)	139(22–341)	0.356
During anti-tuberculosis treatment[median (range)]			
Peak AST	163.5(46–438)	27(11–72)	0.000
Peak ALT	155.5(80–553)	16(5–42)	0.000
Peak TBil	7.25(2.7–44.3)	8(1.2–27)	0.667
Peak ALP	163(70–273)	140(53–378)	0.255
Temporal profile			
Interval between institution of treatment and onset of symptoms in days range (median)	16 (7–26)	-	
Interval between cessation of treatment and normalization of ALT in days range (median)	18 (7–39)	-	

For the GSTM1, 14 children (70.0%) in cases and 96 children (67.1%) in controls had homozygous null mutations, while no patient in cases and 7 children (4.9%) in controls had heterozygous null mutations. For the GSTT1, 13 children (65.0%) in cases and 97 children (67.8%) in controls had homozygous null mutations, 5 children (25.0%) in cases and 37 children (25.9%) in controls had heterozygous null mutations. Neither the GSTM1, nor the GSTT1 polymorphism was significantly correlated with the occurrence of ATHD (Table 3).

**Table 3:** GSTT1 and GSTM1 genotypes in cases and controls.

		Cases	Controls	*P*
*GSTM1*	*0/*0/poor metabolizers	14(70.0%)	96(67.1%)	0.599
	*1/*0/intermediate metabolizers	0(0.0%)	7(4.9%)	
	*1/*1/extensive metabolizers	6(30.0%)	40(28.0%)	
*GSTT1*	*0/*0/poor metabolizers	13(65.0%)	97(67.8%)	0.826
	*1/*0/intermediate metabolizers	5(25.0%)	37(25.9%)	
	*1/*1/extensive metabolizers	2(10.0%)	9(6.3%)	

## Discussion

Our study evaluated for the first time the association between the GSTM1/GSTT1 polymorphisms and risk of ATHD in children. The results did not support the GSTM1 or GSTT1 polymorphisms as risk factors of the development of ADTH in Chinese Han children receiving anti-tuberculosis drug therapy.

The efficacy and safety of anti-tuberculosis drug therapy exhibit large inter-individual variability, even in patients treated with the same kind of drugs and standard dosing regimen. Genetic factors undoubtedly contributed to this diversity, in addition to demographic factors (i.e. age, sex, diet) and clinical factors (i.e. liver and kidney function, diseases, co-medication) [9], [23], [24]. A few studies have investigated the roles of GST polymorphisms on the occurrence of ATDH (Table 4). All of the published studies were conducted in adults and showed controversial results. In Taiwan [25] and Indian [26] studies, the GSTM1 homozygous null mutation, but not the GSTT1, was a risk factor of ATDH. In Spain study [27], the GSTT1 homozygous null mutation, but not the GSTM1,was significantly associated with ATDH. In Korean [28], Brazilian [29], and another Indian studies [30], neither the GSTM1 nor the GSTT1 polymorphisms was significantly associated with ATHD.

**Table 4:** Previously published studies on the association of GSTT1 and GSTM1 genotypes with ATDH.

Population	Total Cases	Total Controls	GSTT1 null mutation (*0/*0)			GSTM1 null mutation(*0/*0)			Definition of ATDH	Reference
			Case (%)	Control (%)	*P*	Case (%)	Control (%)	*P*		
Han Chinese children	20	143	13(65.0)	97(67.8)	NS	14(70.0)	96(67.1)	NS	(1)ALT ≥2 ULN (2)AST>40 IU/L,BIL>19.0 umol/L,ALP>220 IU/L, and one of them ≥2 ULN	Present
Chinesewithin four provinces of China	89	356	40(44.9)	164 (46.1)	NS	55(61.8)	203 (57. 0)	NS	(1)ALT ≥2 ULN (2)AST>40 IU/L,BIL>19.0 umol/L, and one of them ≥2 ULN	[17]
Chinese	104	111	/	/	/	63(60.6)	54(48,6)	NS	(1)ALT ≥2 ULN (2)increased AST/ALT/serum proteins(ALP/BIL),at least one these being ≥2 ULN	[38]
Xinjiang Uyghur Autonomous Region, China	89	2155	18(20.22)	420(19.49)	NS	41(46.07)	792(36.75)	NS	ALT, AST or BIL ≥2 ULN	[39]
Taiwan	63	63	24 (38.1)	44 (38.3)	NS	42(66.7)	29 (46.0)	0.033	(1) AST or ALT ≥5 ULN (2) ALP ≥2 ULN (3) BIL>2.5 mg/dl	[25]
Korea	57	190	34 (59.6)	103 (54.2)	NS	26(45.6)	104 (54.7)	NS	AST or ALT ≥3 ULN	[28]
India	51	100	3 (5.9)	3 (3.0)	NS	25(49.0)	49 (49.0)	NS	(1)ALT ≥3 ULN (2) TBIL>1 mg/dl	[30]
India	33	33	5 (15.2)	1 (3.0)	NS	17(51.5)	8 (24.2)	<0.05	ALT ≥2 ULN andBIL>3.0 mg/dl	[26]
North Indians	55	245	14(24.45)	81(33.06)	NS	19(34.55)	42(17.14)	<0.01	(1) AST or ALT ≥5 ULN (2) ALP≥2ULN withhyperbilirubinaemia	[40]
Western Indian	50	246	11(22.00)	30(12.20)	NS	21(42)	61(24.80)	<0.02	ALT, AST or BIL ≥2 ULN	[41]
Brazilian	26	141	4 (15.4)	27 (19.2)	NS	11(42.3)	61 (43.3)	NS	AST or ALT ≥3 ULN	[29]
Brazilian	59	118	11(18.64)	28(23.73)	NS	21(35.59)	34(28.81)	NS	ALT ≥2 ULN (ALT>42 [IU]/L)	[42]
Spain	35	60	17 (48.6)	16 (26.7)	0.03	12(34.3)	25 (41.7)	NS	AST or ALT≥3 ULN	[16]

Several confounding factors may contribute to theses controversial results. Firstly, the definitions of ATDH in these studies were different and some of them may introduce a selection bias, e.g. limited scope of liver injury index, the bias in the definition of clinical symptoms etc. Secondly, the inclusion and exclusion criteria were different in these studies, and some of them did not exclude other causes of liver injury, thereby, resulting in false positive patients in cases. Finally, the anti-tuberculosis therapeutic regimens were different. It has been reported that the risks of ATHD were not the same between different treatment combinations [14]. These confounding factors have been fully considered when designing the present study. All these conditions allow eliminating the potential impacts of these confounding factors on our results.

ATHD is an idiosyncratic drug reaction, for which reactive metabolite, rather than the parent drug, is responsible [31], [32].Drug metabolizing enzymes have critical effects by both synthesis and detoxification of reactive toxic metabolites. In the liver, isoniazid is firstly metabolized into acetylisoniazid via N-acetyltransferase (NAT), followed by hydrolysis to acetylhydrazine. Acetylhydrazine is then oxidised into hepatotoxic metabolites by CYP2E1. The hepatotoxic metabolites formed through NAT or CYP2E1 are further detoxified by GST. Clinical experience has revealed that children differ from adults in terms of the risk of ATHD. Adverse drug reaction is linked to developmental changes of drug metabolism, so it has age-dependent predisposition [33], [34], which might explain the different impacts of the GST polymorphisms on the risk of ATHD between children and adults. Indeed, The ontogeny of both NAT and CYP2E1 has been reported in children [35]–[37], who had a lower metabolism capacity as compared to adults, thereby, reducing the formation of hepatotoxic metabolites produced by NAT and CYP2E1. As a consequence, the detoxification burden is reduced and the impacts of GST polymorphisms on ATHD might be less important in children as compared to adults.

Our study had some limitations. Due to the small sample size, our study is underpowered to detect a smaller difference than the one expected. In addition, the pharmacokinetic data of parent drugs and metabolites are still missing in Chinese children. Given the ethic difference, the ontogeny data in Caucasian children should be extrapolated with caution to Chinese children. Further research is required to confirm our hypothesis.

In conclusion, in the present case-control study, we evaluated for the first time the impacts of GSTM1 and GSTT1 polymorphisms on the development of ATHD in children. The GSTT1 and GSTM1 null mutations did not increase the risk of ATDH in Han Chinese children. Our results do not support a routine genetic testing of GSTM1 and GSTT1 for ATHD in children.
